# Supplementary figures and images for: Multi-Omic Investigations of a 17–19 Translocation Links MINK1 Disruption to Autism, Epilepsy and Osteoporosis
Source: Int J Mol Sci. 2022 Aug 20;23(16):9392. doi: 10.3390/ijms23169392 (PMC9408972; doi:10.3390/ijms23169392)

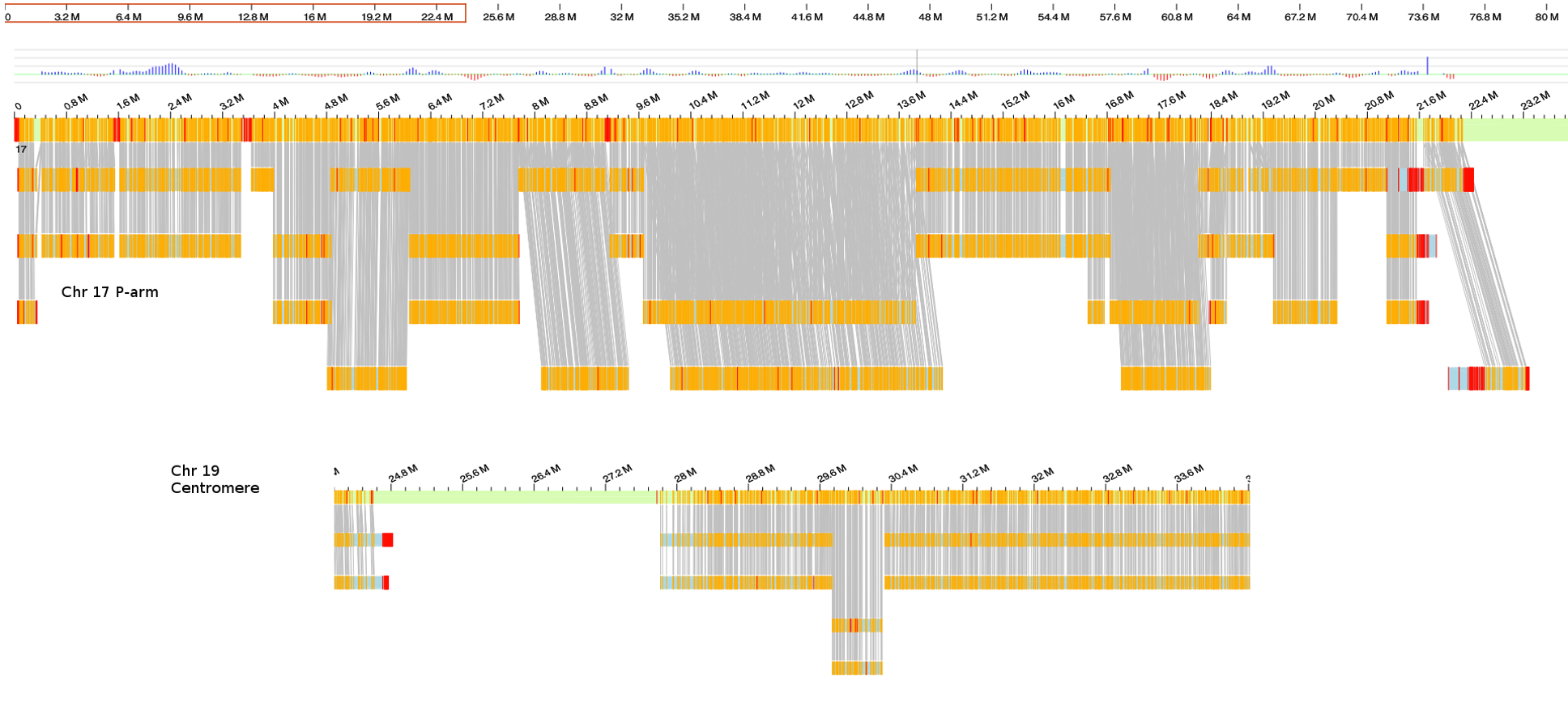

Supplement: Supplementary file 1 [file ijms-23-09392-s001.zip › Figure S1.tiff]
